# Dendritic cells and natural killer cells: The road to a successful oncolytic virotherapy

**DOI:** 10.3389/fimmu.2022.950079

**Published:** 2023-01-10

**Authors:** Matin Ghasemi, Laleh Abbasi, Leila Ghanbari Naeini, Pajman Kokabian, Najmeh Nameh Goshay Fard, Nozar Givtaj

**Affiliations:** ^1^Faculty of Medicine, Tonekabon Branch, Islamic Azad University, Tonekabon, Iran; ^2^Guilan University of Medical Sciences, Rasht, Iran; ^3^Gulf Medical University, Ajman, United Arab Emirates; ^4^School of Medicine, Shahid Beheshti University of Medical Sciences, Tehran, Iran; ^5^Thalassemia & Hemoglobinopathy Research Center, Health Research Institute, Ahvaz Jundishapur University of Medical Sciences, Ahvaz, Iran; ^6^Rajaei Cardiovascular, Medical and Research Center, Iran University of Medical Sciences, Tehran, Iran

**Keywords:** dendritic cells, natural killer cells, cancer, virus, virus immunology, oncolytic virotherapy

## Abstract

Every type of cancer tissue is theoretically more vulnerable to viral infection. This natural proclivity has been harnessed as a new anti-cancer therapy by employing oncolytic viruses (OVs) to selectively infect and destroy cancer cells while providing little or no harm with no toxicity to the host. Whereas the primary oncolytic capabilities of OVs initially sparked the greatest concern, the predominant focus of research is on the association between OVs and the host immune system. Numerous OVs are potent causal agents of class I MHC pathway-related chemicals, enabling early tumor/viral immune recognition and cytokine-mediated response. The modified OVs have been studied for their ability to bind to dendritic cells (DCs) by expressing growth factors, chemokines, cytokines, and defensins inside the viral genome. OVs, like reovirus, can directly infect DCs, causing them to release chemokines and cytokines that attract and excite natural killer (NK) cells. In addition, OVs can directly alter cancer cells’ sensitivity to NK by altering the expression levels of NK cell activators and inhibitors on cancerous cells. Therefore, NK cells and DCs in modulating the therapeutic response should be considered when developing and improving future OV-based therapeutics, whether modified to express transgenes or used in combination with other drugs/immunotherapies. Concerning the close relationship between NK cells and DCs in the potential of OVs to kill tumor cells, we explore how DCs and NK cells in tumor microenvironment affect oncolytic virotherapy and summarize additional information about the interaction mentioned above in detail in this work.

## Introduction

Tumor cells endure many genetic and physiological alterations that separate them from healthy cells during the oncogenic procedure. Tumor cells develop to resist immune-mediated detection and elimination, including acquiring abnormalities in cellular anti-viral pathways, one of these cancer-inherent hallmarks (1). Every type of cancer tissue is potentially more vulnerable to infection by at least certain viruses, and oncolytic viruses (OVs) have been used to selectively infect and destroy cancer cells while providing little or no toxicity to the host in a new anti-cancer treatment (2). In comparison to currently existing medicines, OVs have distinct modes of action. The immune response resulting from the function of OVs involves direct oncolysis of cancer cells or activation and recruitment of immune cells in the tumor microenvironment (TME) (3). At first, it was believed that direct oncolysis was the sole route through which OVs exercised their anti-tumor influence, and that the immune system was the primary obstacle to oncolytic virotherapy. However, recent research has shown that indirect oncolysis can also be effective. Over the course of the past ten years, there has been a significant amount of debate on whether or not the immune system is OVs' friend or foe. The initial position has been rethought, however, as a result of a mountain of evidence demonstrating that the immune system plays a substantial part in the effectiveness of oncolytic virotherapy. This evidence has caused the view to be overturned (4). Different oncolytic viruses eliminate tumor cells by activating a number of distinct cell death pathways, each of which is immunogenic to a different degree. All of them emit pathogen-associated molecular patterns, which leads to an environment that is considered to be "acutely inflamed." This environment is characterized by activated de novo invading and resident dendritic cells (DCs), macrophages, and NK cells, amongst other immune cells (5). These cells have the ability to eliminate malignant cells that have been infected by a virus, create cytokines that delay the progression of tumors, and gather viral and tumor antigens from dead tumor cells in order to deliver them to T lymphocytes and activate them. It is possible that Myeloid-derived suppressor cells (MDSCs) and regulatory T cells (Tregs) will be recruited into the tissue microenvironment (TME) at the same time, which will restrict immune responses (6, 7). Regarding the significant role that NK cells and DCs play in the capacity of OVs to kill tumor cells, the purpose of this paper is to investigate how the presence of DCs and NK cells in the TME affects oncolytic virotherapy and to provide a summary of additional information regarding the interaction in question.

## Development and activity of DCs and NK cells in immune response

### DCs

DCs were first discovered in the skin by Paul Langerhans in 1868, who discovered a population of cells with projections comparable to the dendrites of neurons (8). Nearly a century later, in 1973, Steinman and Cohn identified a cell population in the spleens of mice comparable to Langerhans’. Compared to monocytes and macrophages, these cells had a different cellular appearance and behavior; hence they were dubbed DCs (9). This new cell type was shown to have a high capability for initiating and modulating immune responses. DCs are lymphoid or myeloid lineage cells that originate in the bone marrow and dwell in peripheral and lymphoid tissues. They are engaged in immune surveillance and T cell immune response activation (10). These progenitor cells develop into immature DCs with high endocytic activity but poor T-cell activation capability (11). Immature DCs have a number of pattern recognition receptors that continually scan the surroundings for pathogens like viruses and bacteria (12). DCs are immune system sentinels kept immature and inactive under normal conditions. DCs undertake a multifaceted sequence of morphological and functional alterations known as maturation when exposed to optimum inducements such as inflammatory cytokines, microbial factors, or endogenous alarmins (13). Mature DCs have many phenotypic and functional properties, including the acquiring chemokine receptors (e.g., CCR7), increased expression of adhesion molecules, T cell co-stimulatory molecules (CD80 and CD86), immunoproteasomes, and peptide-MHC class I and II molecules, and the capacity to release several types of cytokines (e.g., IL-12), all of which are required for cell migration to lymphoid (14). Conventional DCs (cDCs), also known as myeloid DCs (mDCs), and plasmacytoid DCs (pDCs), are the two primary subtypes of DCs (15). Pre-cDCs are created in the bone marrow and are used to make conventional DCs. They enter the bloodstream and subsequently seed other tissues. Their development *in vitro* necessitates the presence of GM-CSF and Flt3L, expressing CD1a, CD11c, CD13, and CD33, but not CD14 or CD16 (16). The expression of various toll-like receptors (TLRs) such as TLR1-TLR8 and TLR10 distinguishes conventional DCs. cDCs are split into two types based on the expression of surface markers: CD1c+ mDC and CD141+ mDC (17, 18). Plasmacytoid DCs get their name because they look like plasma cells and are known for producing large amounts of type 1 interferons (IFNs) in response to identifying active or inactivated viruses or by interaction with DNA *via* TLR7 and TLR9. They additionally express TLR1, TLR6, and TLR10 in addition to these TLRs (19).

DCs serve as the immune system’s sentinels, bridging innate and adaptive immune responses (20). DCs are the utmost powerful specialized antigen presentation cells (APCs) since they uptake, process, and present antigens, such as tumor antigens, to trigger naïve antigen-specific CD4 and CD8 T cells and begin all adaptive immune responses (21). In addition, DC are capable of producing cytokines and growth factors, which can alter immune responses over the course of time (22), and the contacts that DC have with other immune cells, such as NK and innate lymphoid cells (ILCs), can influence the behavior of those other immune cells (23, 24).

### NK cells

As the third most common lymphocyte in the blood after T and B cells, NK cells can identify various damaged cells, such as cancer cells and virus-infected cells. The lack of these immune cells has been related to the spontaneous growth of malignancies in mice in genetic trials (25). NK cells, like other cells in the circulatory system, originate from pluripotent hematopoietic stem cells in the bone marrow. These cells then go through a series of maturation and differentiation procedures in order to become mature NK cells (26). Following an intravenous injection, NK cell progenitors begin to express FcR receptor III and begin the process of giving birth to NK cells. During the course of their development, these cells start to express certain surface receptors, such as CD56 and KIRs (in humans) or NK1.1 and Ly49 (in mice) (27, 28). Mature cells take on the appearance of large granular lymphocytes as they mature. These granules, which are responsible for NK cell-mediated death, contain perforin, a protein that disrupts membranes, and granzymes, a family of proteolytic enzymes. Perforin and granzymes are found together in these granules. These granules are exocytosed through the immunological synapse that is formed between NK cells and target cells when these cells connect with one another. This leads to the specific lysis of the target cell (29, 30). The production of cytokines and chemokines by these immune cells in response to stimulation by damaged cells is one way that the process of activation of innate and acquired immune cells can be completed (31). Interleukins (such as IL-10 and TNF) and growth factors (such as GM-CSF and CCL3) can be released by NK cells, as well as chemokines (such as CCL3 and CCL4) (32). Releasing these cytokines attracts additional immune cells to the site of inflammation, causing them to become activated and proliferate. IFN is produced early and powerfully by NK cells. It has a variety of impacts on the immune system, including inducing MHC class II molecules on antigen-presenting cells (APCs), activating myeloid cells, and inducing T cells (33). NK cells are activated by a balance of activating and inhibiting signals derived from germline-encoded receptors. Tolerating NK cells against normal cells depends on a number of inhibitory receptors, such as CD94/NKG2A and ILT2/LIR-1/CD85j. This is accomplished by binding to MHC-I ligands, which are expressed on them (34). Whenever NK cells interact with MHC-I-expressing normal cells, inhibitory receptors send negative signals to the immunological synapse, effectively blocking stimulatory signals from co-engaged activating receptors. Many cancers and virus-infected cells suppress MHC-I expression on their surfaces to avoid being recognized by the antigen receptor on cytotoxic T cells. However, in the nonappearance of tolerizing MHC-I ligands, these aberrant cells are fundamentally vulnerable to NK cell-mediated assault (35). NK cells express the triggering receptors FcRIIIA (CD16), 2B4, NKG2D, and NCR, also identified as NKp30, NKp44, and NKp46 (36). The NCR and NKG2D receptors are principally critical for activating NK cell responses against targeted cancerous cells (37). Activation of the activating receptor NKG2D, in contrast to inhibitory receptors, is a fundamental procedure by which NK cells detect stressed or sick cells and kill them (38). In humans, NKG2D identifies MHC chain-related (MIC) A, MICB, and UL16-binding proteins (ULBPs), which are HLA-related molecules that lack peptide presentation capability and are upregulated in stressed cells, such as cancers (39). The relative expression of the adhesion molecule CD56 and the Fcγ receptor CD16 distinguishes various NK cell subsets in humans. The CD56^bright^CD16^-^ and CD56^dim^CD16^bright^ subpopulations are the most well-studied. The latter is in number, dominant in peripheral blood and is usually the utmost cytotoxic subtype, whilst the former especially produce cytokines (40, 41). Whereas the majority of publications argue that CD56^bright^ NK cells are the juvenile antecedents of the CD56^dim^ subgroup, this has not been shown conclusively, and the idea of two distinct lineages has not been ruled out entirely (42). According to a recently projected classification (43), based on their granule content and differential expression of various surface markers and transcription factors, circulating CD56^bright^ NK cells, canonical CD56^dim^ NK cells, adaptive CD56^dim^ NK cells, and tissue-resident CD56^bright^ NK cells are separated into four subgroups (44).

NK cells can also potentially become memory cells, which can be thought of as adaptive immune cells. In addition, these immune cells can directly or indirectly (by producing cytokines) affect the function of adaptive immune cells (45, 46). As a result, NK cells play a crucial part in immune responses to malignancies and viral infections. Furthermore, a better knowledge of the processes that drive NK cell activation has given rise to the progression of therapeutic drugs that can boost their responsiveness.

## DCs and NK cells in the TME

Alterations in the stroma that surrounds a cancerous tumor have been related to both the development and progression of the disease. Cancer cells have the potential to exert control over their immediate surroundings thanks to the creation of a wide variety of cytokines, chemokines, and other chemicals (47). The cells in the surrounding area undergo reprogramming, which subsequently enables those cells to play an essential role in the survival and growth of the tumor. Immune cells are an essential component of the stroma of the tumor and have a substantial impact on the outcome of this process. When both innate and adaptive immune cells are present in the TME, there is mounting evidence suggesting that these cells contribute to the progression of the tumor (48–50).

### DCs

Antigen presentation suppression is common for malignancies to evade the immune system (51). DCs, according to their function in immune cells, are expected to be able to boost antitumor immunity due to their status as “professional” APCs. Nevertheless, as cancer advances, DCs fail to stimulate anti-tumor immunity, resulting in immunological repression. There are several conditions that must be met for antigen presentation to be effective at stimulating antitumor T cell responses, such as a) obtaining and processing tumor antigens with the right DC, b) processing antigens efficiently and delivering pMHC to DC surfaces, and c) augmenting DC co-stimulatory/homing molecules so that T cells can be activated effectively (52). Regrettably, cancerous tumors can affect DCs at each stage through several methods that either prevent the production of tumor-associated antigen-specific T lymphocytes or encourage immune cell tolerance to the tumor (53). In addition, tumors can evade the immune system by keeping DCs immature, preventing them from stimulating an anti-tumor T-cell response. Tumor-derived factors (such as IL-6, IL-10, and VEGF) impede DC maturation and, as a result, their ability to serve as APCs (54).

IL-6 derived from tumors reduces DC maturation and migration, influences the differentiation of DCs into macrophages, and promotes tolerogenic phenotypes in DCs (55). Tumor cells, MDSCs, tumor-associated macrophages (TAMs), DCs, and Tregs, have all been found to generate IL-10 in the TME. DC function has been suppressed by IL-10, inhibiting several parts of DC biology, including DC maturation, their capacity to secrete IL-12, antigen presentation, and T cell priming (56, 57). IL-10 has also been demonstrated to transform immunogenic DCs into tolerogenic DCs, resulting in the production of anergic CD8 T cells that are cytotoxic (58). TME also manipulates the DC function to skew T cell development to avoid immune detection. TME factors, including Matrix metalloproteinase 2 (MMP-2) and Thymic Stromal Lymphopoietin (TSLP), have been demonstrated to influence DC activity and trigger harmful Th2 responses (59, 60). Numerous cancer-related signaling pathways, including β-catenin, MAPK, and STATs, are important in interacting between tumor cells and DCs in the TME (61, 62). T cells and DCs are not recruited into tumors when β-catenin signaling is inhibited in melanoma cells (63), and tumors stimulate the production of “mature” DCs with immunosuppressive rather than immunostimulatory characteristics. Pattern recognition receptor (PRR) ligands do not activate Regulatory DCs, a DC subgroup that is dependent on the transcription factor Satb1. Instead, inflammatory mediators such as IL-1β, TNF-α, type I IFN, and prostaglandin E2 activate Regulatory DCs (64).

### NK cells

NK cells affect the function of other innate and adaptive immune groups in the TME or interact directly with tumor cells to affect tumor formation. In many experimental mouse tumor models, the role of NK cells in antitumor immunity has been proven (65). The majority of this research included implanting syngeneic cancer cells into mice that were either genetically defective in NK cell activity or had their NK cells depleted by antibodies. In these mice, removing NK cells resulted in more aggressive tumor development and metastasis (66). Most of the evidence regarding NK cells’ participation in tumor monitoring in people comes from correlative studies. Low NK-like cytotoxicity of peripheral blood lymphocytes was shown to be associated with an elevated risk of cancer in an 11-year follow-up study (67). Also, many studies have since discovered that high numbers of tumor-infiltrating NK cells are linked to a better prognosis in individuals with colorectal cancer, gastric cancer, and squamous cell lung cancer (68). The NK cell lineage has been studied for cancer eradication because of its capacity to spontaneously destroy a wide variety of tumor cells while sparing normal cells. In contrast to T cells, which APCs must first educate, NK cells detect prospective target cells without the necessity for vaccination or pre-activation (69). NK cells can also detect malignancies that may elude T-cell destruction due to abnormal HLA (human leukocyte antigen) expression.

In addition, the production of NK cell ligands by malignant cells and the activation of NK cells helped the adaptive response of tumor-specific T-cells and decreased the formation of tumors in a mouse model of lung adenocarcinoma that was developed relatively recently (70). These findings, together with previous research, suggest that NK cells may have an indirect role in modifying the immune response in the TME. Because of this, it is imperative that novel immunotherapies based on NK cells be developed (71). In addition, cancer cells have devised several methods to evade being detected by NK cells, which is more evidence that NK cells play an important role in the control of tumors. TGF-β, IL-10, prostaglandin E2, indoleamine 2,3-dioxygenase (Ido), and adenosine are examples of immune-suppressive substances that tumor cells can upregulate or release (72, 73). Another potential technique used by tumor cells to lower the number of activating ligands on their surface and/or promote NK cell desensitization is the shedding of ligands for activating receptors (33, 74). The inability of NK cells to reach the lesion because of imperfect vascularization, the undersupply of expression of adhesion molecules, the elevated expression of MHC class I on tumor cells, resistance to Fas- or perforin-mediated apoptosis, and the release of immunosuppressive factors by tumors, such as IL-10 or TGF-β, are some of the other mechanisms that have been proposed (75). Enhancing cytotoxicity or restoring the immune system is one of the necessary steps that must be taken before attempting to effectively increase cancer immunotherapy. The primary goal of immunotherapy is to "push" immune activation by using additives such as cytokines and antibodies to regulate the processes that increase the number and/or quality of anti-tumor immune responses. This is accomplished via the use of the phrase "push." Cytokines have an important role in the processes of lymphocyte survival, proliferation, differentiation, and activation. IL-2, IL-15, IL-12, IL-21, and IL-18 all boost anti-tumor efficacy and increase NK cell proliferation when tested in vitro as well as in vivo. In addition, it has been established that monoclonal antibodies may directly and/or indirectly augment the activities of NK cells in vivo, making them appropriate for the treatment of cancer using immunotherapy (76).

### Interaction of NK cells and DCs

The interplay between NK cells and other immune cells in the TME, such as T-cells and DCs, starts significant anti-tumor actions and their capacity for directly eradicating cancer cells. This interaction between NK cells and DCs might encourage DC absorption of tumor antigens in secondary lymph nodes, where they are presented to and activated by T cells, allowing for further anti-tumor responses (77). The chemoattractants CCL5, XCL1, and XCL2, secreted by NK cells, have recently been demonstrated to attract classical type 1 DCs (cDC1s), and the amount of this process corresponds with cancer patient survival (78). NK cells and cDC1s interact in both directions, and NK cells and T cells can draw cDC1s into tumors by secreting chemokines, which helps to boost anti-tumor immunity (78). cDC1s are concentrated in TMEs with a particular chemokine profile in both preclinical and clinical situations. XCL1 is mostly secreted by tumor-resident CD56low NK cells, CCL4 and CCL5 are primarily generated by CD56low and CD56high NK cells, and CD8+ T cells are all part of this profile (78). XCR1 and CCR5, which are the receptors for those chemokines, are expressed by cDC1 (78, 79), showing that NK and CD8+ T cells are essential for cDC1 intratumoral migration because XCL1, CCL5, and CCL4 (78). Based on the expression of CCR5, NK cells that have been activated with IL-18 and IFN-α draw in immature DC and cause DCs to produce more CXCL9, CXCL10, and CCL5 to attract effector cells (80). PGE2 reduces the expression of XCR1 and CCR5 on cDC1s *in vitro*, which reduces responsiveness to XCL1 and CCL5. PGE2 also prevents NK cells from secreting XCL1 and CCL5, highlighting its function as an immunosuppressive mediator to prevent cDC1s from migrating to the tumor. CCL5 and XCL1 gene expression and CD8+ T cell infiltration correlated with the expression of the cDC1 and NK cell gene signatures in human malignancies. Additionally, the TME’s NK cell and cDC1 gene signatures have a good correlation with patient survival (81).

## DCs and NK cells against viral infections

Several agents may be detected and employed to stimulate the innate immune system during an infection. These may be divided into pathogen-associated molecular and damage-associated patterns (PAMPs and DAMPs, respectively) (82, 83). The recognition of PAMPs or DAMPs by immature DCs causes their stimulation and subsequent maturation (84). There is a correlation between this event and changes in the morphology and function of DCs, as well as an increase in the expression of co-stimulatory and adhesion molecules (85). Antigens generated by infections are subsequently collected, processed, and presented to naïve T lymphocytes as peptides bound to MHC-II on the surface of DCs. This process occurs after the antigens have been created by infections. It is believed that DCs play a crucial role in the clearance of viral infections. It is necessary to produce significant quantities of cytokines, such as type I IFN, in order to accomplish this clearance (86). The utmost prolific IFN-I-producing cells, plasmacytoid dendritic cells (pDCs), swiftly respond to murine and human persistent viruses by generating large quantities of these cytokines (87). Mice lacking in pDCs consistently fail to manage a variety of acute and chronic viral infections (88). Prophylactic pDC activation temporarily halted IFN-I downregulation, or IFN-I injection before or after infection with a persistent virus improves T - cell activation and host resistance, suggesting rapid and dramatic IFN-I attenuation throughout chronic viral infection enhances T cell exhaustion and viral persistence (89). TLR9 detects the type A CpG oligonucleotides (CpGA) in plasmacytoid DCs (pDCs), encouraging MYD88 to interact with TRAF6 and activate IRF7. TRAF6 activates IRF7 through ubiquitin E3 ligase activity. IRF7 may phosphorylate independently of TBK1/IKK once activated, and it can be translocated into the nucleus to enhance type I IFN-α/β (90, 91). Another TLR involved in antiviral responses is TLR3, found in cDC endosomes and capable of detecting dsRNA (92). TLR3 interfaces with the adaptor protein TIR-containing adaptor molecule-1 (TICAM-1/TRIF) after identifying the dsRNA. This connection activates AP-1, IRF3, and NF-κB, which are then translocated into the nucleus and stimulate the production of IFN-β. Furthermore, stimulation of the NF-κB pathway permits pro-inflammatory genes to be expressed (93). Ultimately, TLR4, found on the surface of cells, may recognize distinct viral proteins, causing the MYD88 pathway to signal and activate type I IFN genes (94). TLR4 can also influence the stimulation of type I IFN genes through other TRIF-related mechanisms, such as when TLR4 interacts with TRIF *via* TRAM, resulting in a late stimulation of the MYD88 pathway and the production of type I IFN genes in response to viral infection (95). TLR2, likewise found on the cellular membrane, has been linked to the detection of viral peptides in the same way as TLR4 has (96).

NK cells are a kind of innate lymphocyte that act as the body's first line of defense against viral infections and malignant cells (97). As part of the innate immune system, NK cells are lymphocytes. NK cells, like T and B cells, do not include antigen-specific receptors. This discovery fueled the hypothesis that NK cells mediate nonspecific cytolysis, first found as a result of their ability to eliminate tumor cells without the need for prior sensitization (98). While their anti-cancer properties were first discovered, NK cells are essential for managing some illnesses, notably viral infections. NK cells show an essential role in the innate immune response to viruses in humans, including herpes viruses, poxvirus, and human papillomaviruses (99). NK cells employ various techniques to detect inflammatory signals during viral infection. First, they express receptors for cytokines, including IFN-α, IL-12, IL-15, and IL-182, whose expression is significantly upregulated early upon infection and which play a critical role in activating the NK cell pool and facilitating virus protection, as best investigated in the murine cytomegalovirus (MCMV) infection model (100). The majority of NK cells express the vast bulk of cytokine receptors. Proinflammatory cytokines signal with most, though not all, NK cells and activate the whole NK cell compartment due to this essentially uniform expression pattern, indicating that the cytokine–cytokine receptor axis plays a fundamental function in stimulating NK cells (101). Type I IFN signaling through STAT1 and IL-12 and IL-18 signaling *via* STAT4 culminate in remarkably diverse interactions with the NK cell epigenetic landscape to promote early NK cell activation. Immediately after NK cell activation, STAT1 and STAT4 can antagonistically affect their downstream pathways, with STAT4 actively inhibiting STAT1 expression (102). As the second mechanism, some NK cell receptors can detect ligands on infected, altered, or stressed cells’ surfaces. In a process known as antibody-dependent cell-mediated cytotoxicity, the Fc receptor Fcγ RIIIa (CD16) on NK cells identifies the Fc part of antibodies, for instance, linked to sick cells, and activates NK cells (103).

Although STAT4 binding improved chromatin accessibility, STAT1 interacting with promoter areas was more prominent. Even though the long-term ramifications of these changes in the chromatin landscape are unknown, remodeling through IL-12 combined IL-18 and STAT4 may have more persistent effects since STAT4 signaling is required to develop both adaptive and cytokine-induced memory-like NK cells (104, 105). Direct Toll-like receptor (TLR) activation on NK cells, in addition to NK cell-activating receptors, has been recognized as a dominant factor in NK cell activation during infections. Human NK cells express TLR3, 7, 8, and 9; ligands for these TLRs have been shown to activate human NK cells *in vitro* (106). The effective control of viruses not only involves the correct activation of NK cells, but also the effective recruitment of NK cells that have been activated to the site of infection (107). NK cells could be seen in the spleen, lung, bone marrow, lymph nodes, and peripheral blood mononuclear cells when the body was in its steady-state condition. Sphingosine-1-phosphate is essential for lymphocyte migration, and S1P5R, a G-protein-coupled receptor (GPCR), is differentially expressed on NK cells during various stages of maturation and in the stable equilibrium to regulate their mobility (108). NK cells travel to and concentrate at infected sites for a variety of viruses, such as LCMV, MCMV, etc., during infection (109). There are four different chemokine receptors related to NK cell migration to inflammation sites. These receptors are CCR2, CCR5, CXCR3, and CX3CR1 (107). Apart from CX3CR1, all have been proven to have a contribution to viral infection chemotaxis.

On the other hand, the mechanisms that are involved in the migration of NK cells are only starting to be identified. The PI3K pathway, and in particular the p110γ and p110δ isoforms, plays an important role in the overall process of leukocyte motility. These two isoforms of PI3K drive NK cell migration in a manner that is very similar, with p110γ signaling downstream of GPCRs in response to chemokines and p110δ signaling downstream of tyrosine kinase-linked receptors in response to other steady-state or proinflammatory signals. NK cell migration is driven by chemokines and proinflammatory signals (110). In spite of this, there has been little investigation into the role that these PI3K isoforms play in the process of NK cell translocation in response to viral infection. The generation of cytokines, the release of cytolytic granules, and the usage of death receptor-mediated cytolysis are the three basic methods that natural killer (NK) cells use to eliminate virally infected cells after they have been activated and attracted to the site of the infection, respectively (111). NK cells, which were once known as "giant granular lymphocytes," are capable of destroying infected cells directly by enabling cytolysis via produced granules. In addition, NK cells were formerly known as "giant granular lymphocytes" (109, 111). These granules, particularly perforin and granzymes, are the same as those seen in cytotoxic T lymphocytes, and they accomplish their mission by interacting directly with infected cells. In addition, NK cell-mediated cytolysis of infected cells may also make use of FasL and apoptosis-inducing ligand related to tumor necrosis factor (TNF) (69). NK cells are responsible for the production of ligands that activate death receptors on the target cell. This in turn causes the extrinsic process of apoptosis to be activated.

## DCs and NK cells as the enhancer of OVs

Whereas the primary oncolytic properties of OVs received the most concern initially, there is currently a rising interest in how OVs interact with the immune system. Because the immune system cannot make a distinction between therapeutic and pathogenic viruses, OVs are challenged by the immune system’s effort to clean them out of the body. The adaptive immune response’s defensive activity is generally initiated by circulating virion-associated antigens or cell-associated viral gene products, which are produced when target cells are infected with a virus. Specialized immunoglobulin (Ig) surface receptors on B cells identify these antigens, causing these cells to become activated and produce neutralizing anti-viral antibodies (112).

The natural role of DCs, which is to present tumor antigens, is absolutely necessary for the development of anti-tumor immunity. DCs are able to interact with a wide variety of immune cells, which allows them to start and sustain both innate and adaptive immune responses (113). However, various immunosuppressive mechanisms present in the TME decrease DC function and impede the development of anti-tumor immunity (114), which reduces the efficacy of immunotherapy. As a consequence of this, efforts are being undertaken to prevent the immunosuppression that is linked with tumors. Anti-cancer treatment based on oncolytic viruses is one of the most fascinating therapeutic techniques for addressing such challenges (oncotherapy). OVs selectively target and destroy cancer cells through direct oncolysis and antitumor immunity (115). Immunosuppression inside the tumor microenvironment (TME) provides an environment that is susceptible to infection and is permissive to viral multiplication, in the end, this leads to death of cancer cells. Upon OV-induced immunogenic cancer cell death and the subsequent "danger" signals, there is an increase in APC and lymphocyte infiltration in the TME. This rise in APC and lymphocyte infiltration leads to the stimulation of nonspecific and specific anti-tumor response, reducing the risk of cancer recurrence over the long term (116). OVs are also responsible for the activation of additional immune responses, which serve to counterbalance the decrease in the presentation of tumor antigens and to boost the interaction of APCs with T cells that are specific to the tumor (117). For instance, several soluble substances released in the TME, including IL-6, IL-10, IDO, M-CSF, transforming growth factor-β1 (TGF-β1), PGE2, and VEGF, can assist the immunosuppression of tumor-infiltrating DCs (118). IL-6 knockout mice (119), anti-VEGF antibodies (120, 121), and anti-IL-8 monoclonal antibodies improved immune control in a series of animal models, resulting in an improved immune response to tumors (122, 123). However, pro-inflammatory cytokines are also present in the TME, which helps to improve DC maturation, enhance antigen priming, and boost immune infiltration in malignancies (118). Consequently, the intricate balance of inflammatory signals in the TME is a topic of significant study interest but is currently difficult to focus. In a recent study on human melanoma, pro-inflammatory cytokine FLT3L production (by NK cells) was linked to an abundance of intratumoral stimulatory DCs, greater patient response to anti-PD-1 treatments, and higher overall survival (124). As an invading pathogen, OV exposure causes a rise in the production of type I IFN and a subsequent pro-inflammatory immune response to restrict and eliminate the virus. Numerous OVs are powerful causative agents of class I MHC pathway-related molecules (125), allowing for prompt tumor/viral immune detection in addition to a cytokine-mediated response. Investigations on modified OVs such as adenovirus, HSV, and others have concentrated on expressing growth factors (GM-CSF and Flt3L), chemokines (CCL2), cytokines (IL-12, RANTES, and IFN-β), and defensins (β-defensin-2) within the viral genome to increase the connection of OVs with DCs. The introduction of an E1B-deficient oncolytic adenovirus expressing β-defensin-2 (Ad-BD2-E1A), for example, boosted type I IFN responses within the TME by attracting and activating pDCs preferentially (126).

In addition, an oncolytic adenovirus that expresses MIP-1 and Flt3L was developed in order to boost DC recruitment and proliferation in vivo. This resulted in a large synergistic influence on the DC and T cell infiltration of tumors (127). In B16-F10 melanoma tumor-bearing mice, the treatment of IL-12 and GM-CSF-expressing adenovirus (Ad-B7/IL12/GMCSF) in conjunction with DCs resulted in enhanced DC migration to regional lymph nodes due to overexpression of CCL21+ lymphatic arteries around cancer tissues (128). When OV vaccines stimulate DCs, they may become more effective. Infection of follicular B cells in the spleen occurs after systemic delivery of the OV vaccine, which is a rather unusual way to vaccinate. This type of infection is a failed one because virally encoded proteins (including DCT) are only transiently produced. This antigen is transmitted to DCs and then delivered to memory T cells in the central memory T cells (TCM). These APCs live in a privileged location because effector T cells (TEFF) do not penetrate the splenic follicle. This enables the oncolytic boost to be given soon after the priming vaccine, which is critical for treating metastatic cancer patients who do not have time to wait for the TEFF cells created during the priming vaccination to clear. This unique biology underlying the significant development of anti-tumor immunity might be found by using an OV as a vaccine vector that is given intravenously (129). Zhang et al. evaluated the effectiveness, safety, and immunomodulatory impact of intraperitoneal injection of human type 5 recombinant adenovirus (referred to as H101) versus malignant ascites in a recent study in 2022. At baseline, days 7 and 14, and days 14 and 21 after treatment, mass cytometry and immunocytological examination revealed that intraperitoneally injecting H101 significantly reduced tumor cell density and boosted dendritic cell and CD8+ T cell densities (130). Liu et al. also examined the anti-tumor function of the conditionally replicating adenovirus serotype 5 (CRAd5) encoding tumor necrosis factor-related apoptosis-inducing ligand (TRAIL) in several murine tumor models. They discovered that TRAIL expression increased or activated tumor-infiltrating T cells. Mouse CD40 ligand (mCD40L), an immune activator generated *via* recombinant Ad5 vector, was initially utilized in combination with CRAd5-TRAIL for tumor immunotherapy to further enhance the anticancer effects. Studies conducted both *in vitro* and *in vivo* showed that mCD40L efficiently stimulated DCs, B cells, and tumor-infiltrating T cells.

Oncolytic adenoviruses were significantly more effective in CT26 and B16 tumor models when TRAIL receptors were boosted, which accelerated tumor cell apoptosis (131). Another study compared Talimogene laherparepvec (T-VEC), a herpes simplex virus type 1 based OV, against other previously identified immunogenic cell death (ICD)-inducing drugs (such as doxorubicin) and non-ICD inducing agents to determine T-capacity VEC’s to cause ICD (cisplatin). They also investigated T-capacity VECs to promote human BDCA-1+ myeloid DCs (myDCs) maturation. Last but not least, it was shown that T-VEC therapy has both direct and indirect anti-tumor properties this approach causes tumor cell death at the same time as the release of ICD hallmark mediators and simultaneously promotes the maturation of BDCA-1+ myDCs (132). T-VEC was the first FDA-approved OV for cancer therapy because it was created to selectively proliferate in cancerous cell and increases the production of granulocyte-macrophage colony-stimulating factor (GM-CSF) to boost systemic anticancer immune responses (133). In the context of adoptive T cell therapy, Santos et al. (2018) tested the security and effectiveness of an adenovirus treatment with a lymphodepleting regimen combining cyclophosphamide and fludarabine (ACT). A Syrian hamster pancreatic tumor model (HapT1) injected with tumor-infiltrating lymphocytes was employed to study the replication of the human adenovirus (Ad5/3-E2F-D24-hTNF-α-IRES-hIL-2; TILT-123).

In contrast to lymphodepletion, using the oncolytic virus increased efficacy and survival. Using an immunocompetent mouse melanoma model (B16.OVA) infused with ovalbumin-specific T (OT-I) cells, immune cells responding to TNF-α IL-2 were investigated. Interestingly, the use of an adenovirus promoted tumor control along with elevated intratumoral Th1 cytokine levels, CD8+ T cell infiltration, and CD86+ DC infiltration (134).

**Figure 1:**
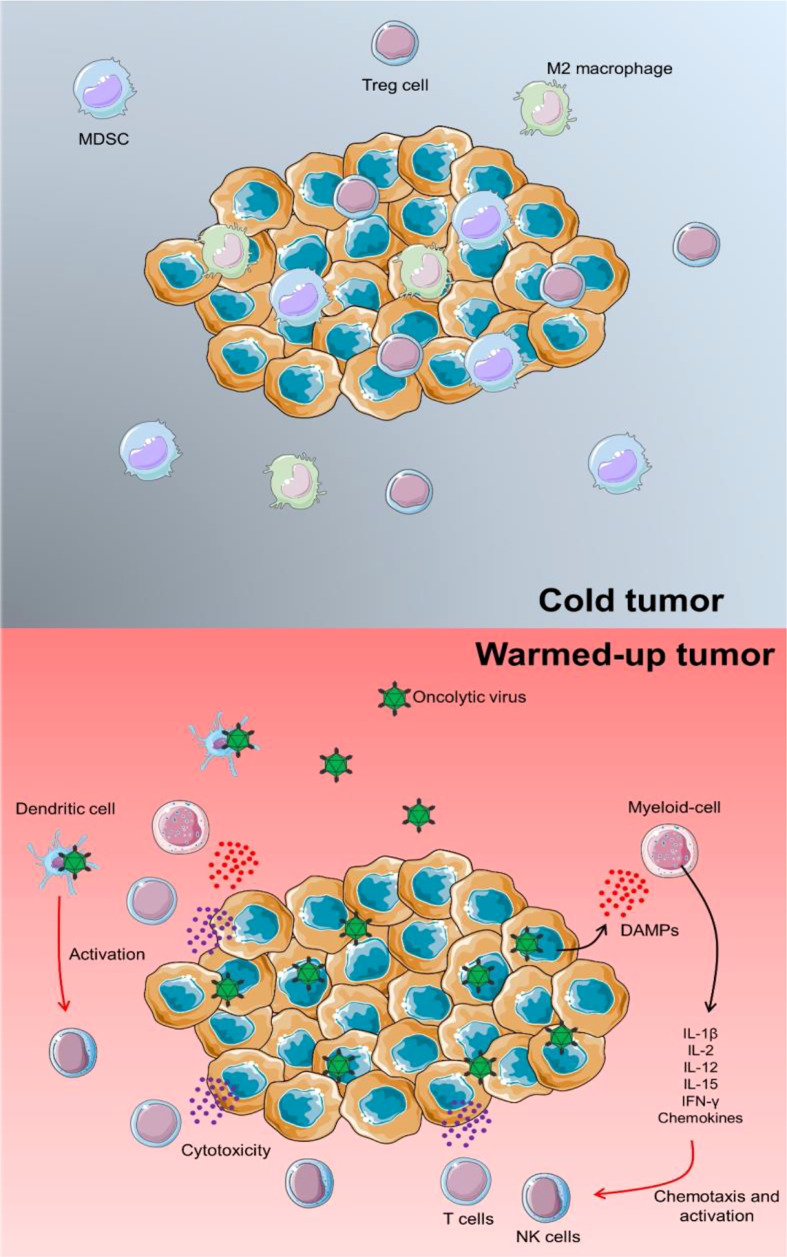
TME warming up *via* OV infection. In a cold tumor, several immunosuppressors could inhibit the proper anti-tumor activity of immune cells. In this case, tumorigenicity could be progressed and lead to the development of other cancer features. Proceeding an OV infection, releasing pro-inflammatory cytokines and chemokines could activate chemotaxis of lymphoid cells such as T cells and NK cells to enhance the immune response against tumor progression. Besides, by direct infection of DCs *via* OVs, these immune cells could trigger NK cells and support a specific cytotoxic response.

NK cells have a role in virus-induced inflammation by targeting and destroying virus-infected host cells as well as producing IFN-γ. As a result, they may negatively impact OV therapy by inhibiting OVs from spreading intra-tumoral and restricting the degree of virus-mediated oncolysis. This important aspect of OVs becomes even more critical when it is realized that immunotherapies frequently fail due to the TME’s immunological “cold” condition. Immunosuppressive cells, including Tregs, MDSCs, and M2 macrophages, are abundant in cold tumors, dampening immune responses. OVs have the advantage of “warming up” immunologically cold tumors to the level of an immunologically activated malignancy (**Figure 1**).

This process begins with the first step, which is the immunogenic cell death of OV-infected tumor cells, which is accompanied by the production of DAMPs and viral products. These compounds have a potent stimulating effect on cells of the innate immune system, particularly those of the myeloid lineage (135). Immune cells in the TME, particularly NK cells, are attracted and stimulated when myeloid cells secrete Immune cells in the TME, particularly NK cells, are attracted and stimulated when myeloid cells secrete cytokines and chemokines that promote inflammatory cytokines. OVs can directly infect DCs such as reovirus, which then causes them to secrete chemokines and cytokines that attract and stimulate NK cells as well as detect DAMPs (136, 137). Besides the extensive immunological effects of inflammation, oncolytic viruses modify the expression of activating and inhibitory NK ligands in cancer cells, thereby increasing their susceptibility to NK cell death. For instance, adenoviruses stimulated the production of NKG2D ligands (138) and influenza-infected prostate cancer cells produced viral hemagglutinin (HA), which binds to NKp46 (139), both of which increased tumor cell vulnerability to NK death. Downregulation of MHC I molecules enhanced tumor sensitivity to NK death, as demonstrated *in vitro* and *in vivo* in glioma cells infected with the Myxoma virus (140). OVs differ from previous immunotherapies in that they can transport therapeutic payloads directly into the TME, triggering the activation of NK cells. Although cytokine injections have the ability to activate dormant natural killer (NK) cells, the therapeutic advantages for patients are severely limited due to the short half-life of the molecules involved and the toxicity of the infusions themselves (141). As a result, utilizing OVs to transfer cytokines to the TME is a viable option. The following cytokines might be used as examples: IL-2, IL-15, IL-12, IFNs, and GM-CSF (142). OV-based vaccinations have recently been tested as anti-cancer medicines. It was discovered, for instance, that vaccination with tumor cells that had been infected ex vivo with a Maraba oncolytic virus expressing IL-12 triggered DC synthesis of CXCL10, which subsequently attracted IFN-γ generating NK cells in the TME. This was accomplished by triggering DC creation of CXCL10 (143).

Similarly, an adenovirus expressing CCL5 as a vaccination increased the number of innate immune cells, including NK cells and cytotoxic T cells, in the TME (144), demonstrating the significant ability of these treatments to stimulate NK and T cell responses. According to a recent study by Wantoch et al., oncolytic reovirus induces STAT1 and STAT4 activation in both CD56dim and CD56bright NK cell subsets, activating NK cells in a way that is type I interferon (IFN-I) dependent. With separate responses in the CD56^dim^ and CD56^bright^ fractions, gene expression analysis showed the dominance of IFN-I responses. In addition, it indicated activation of genes linked to NK cell cytotoxicity and cell cycle progression. Reovirus therapy, however, lowered AKT signaling and suppressed IL-15-induced NK cell proliferation in an IFN-I-dependent manner. Human CD56^bright^ and CD56^dim^ NK cells both responded to reovirus treatment *in vivo* with similar kinetics, but CD56^bright^ NK cells briefly disappeared from the peripheral circulation during the height of the IFN-I response, suggesting that they had been redistributed to secondary lymphoid tissue (145). It is also said that a novel form of UV-inactivated oncolytic herpes simplex virus type 2 (UV-oHSV2) potently stimulates human peripheral blood mononuclear cells, increasing anticancer activity both *in vitro* and *in vivo*.

Further research revealed that the Toll-like receptor 2 (TLR2)/NF-κB signaling pathway is used by UV-oHSV2-stimulated NK cells to release IFN-γ and exert antitumor activity. For the first time, it was discovered that UV-oHSV2 activation increases the expression of two checkpoint molecules, NKG2A (on NK cells) and HLA-E (on tumor cells). Treatment with anti-NKG2A and anti-HLA-E might improve the anticancer effects of UV-oHSV2-stimulated NK92 cells both *in vitro* and *in vivo* (146). Ma et al. produced OV-IL15C, a herpes simplex 1-based human IL15/IL15Rα sushi domain fusion protein, as well as commercial EGFR-CAR NK cells, and examined their efficiency as a monotherapy and in combination *in vitro* and several glioblastoma (GBM) mice models. From OV-IL15C-infected GBM cells, soluble IL15/IL15Rα complex was released *in vitro*, promoting GBM cytotoxicity and enhancing NK and CD8+ T cell survival. Unlike NK cells transduced with an empty vector, frozen, easily accessible off-the-shelf EGFR-CAR NK cells demonstrated improved killing of tumor cells. Compared to parental OV, OV-IL15C dramatically reduced tumor growth *in vivo* and lengthened the survival of mice with GBM. In an immunocompetent model, OV-IL15C plus EGFR-CAR NK cells synergistically inhibited tumor growth and markedly increased survival compared to either monotherapy. These effects were connected to a larger intracranial infiltration, activation of NK and CD8+ T cells, as well as an increased persistence of CAR NK cells (147). Additionally, Kim et al. created a new oncolytic HSV-1 (Δ6/GM/IL12) co-expressing GM-CSF and IL-12 and assessed its effectiveness against a B16-F10 murine melanoma model in a different study. Compared to therapy with Δ6/GM or Δ6/IL12 expressing either cytokine alone, the injection of Δ6/GM/IL12 reduced tumor development and lengthened survival. In mice treated with Δ6/GM/IL12, flow cytometry and histological examination revealed enhanced CD4+ and CD8+ T cell activation. In mice treated with IL6/GM/IL12, an increase in the phenotypically described IFN-γ-producing cell population was detected using an enzyme-linked immunosorbent spot test. Additionally, Δ6/GM/IL12 generated a B16-F10-specific cytotoxic immune response that boosted CD3+CD8+ T cell production of IFN-γ (148). In three separate surgery-induced metastatic models of cancer, it has been shown that novel Vaccinia virus (VV), VV “TK “N1L (with deletion of both thymidine kinase (TK) and N1L genes) armed IL-12 can considerably prolong postoperative life. Increasing the number of circulating NK cells, made possible by virus-induced cytokine production from cells infected with N1L-deleted VV but not N1L-intact VV, was crucial for effectiveness. This impact was further amplified by providing VV “TK “N1L with IL-12, a strong anticancer cytokine. Before surgery, five daily doses of VV “TK “N1L-IL12 significantly increased postoperative survival. Human IL-12 administered to Syrian hamsters with VV “TK “N1L fully reduced tumor recurrence in surgical models of head and neck cancer (149). Overall, those findings show that transgenic OVs have the potential to improve NK cell responses. As we continue to untangle the complexities of the TME and identify novel therapeutic targets to modify immunological and NK cell responses, exciting new breakthroughs will undoubtedly emerge.

**Figure 2:**
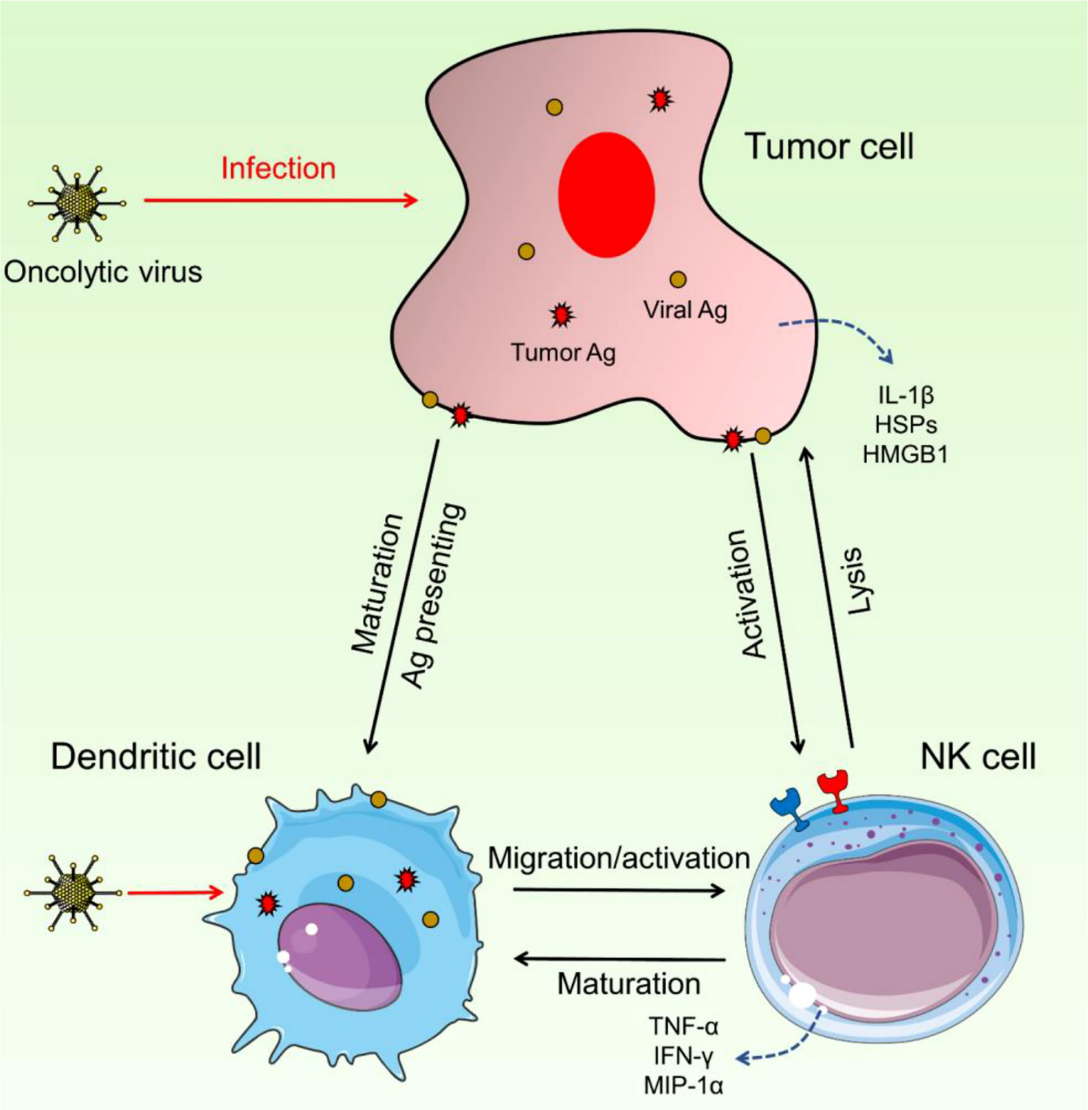
Interaction between NK cells and DCs. These two immune cells could have intermodulation and enhance the activation and migration of NK cells as well as DC maturation. Upon OV infection, DCs can increase the activity and migration of NK cells and on the other side, NK cells trigger the maturation of DCs. Hyperactivated NK cells detect pro-inflammatory cytokines and increase the immune response against cancerous cells.

Intermodulation between NK and DCs appears to be key in the efficacy of OV treatment, which seems dependent on both the innate and adaptive arms of the immune system (**Figure 2**). *In vitro* and *in vivo* studies of NK cell–DC interactions have been conducted. Some OVs can enhance this reaction by causing infected tumor cells to die immunologically, resulting in DC maturation and, most likely, NK cell activation.

NK cells were activated more effectively by DCs made from melanoma oncolysates infected with reovirus than by malignant cells infected with reovirus. An increase in IFN-γ and chemokine production was established by contact-dependent stimulation. By producing type I IFN, DC-MelReo cells matured and NK cells were activated (137, 150). In a mouse model of prostate cancer, oncolytic treatment was dependent on DCs for chemoattractant synthesis, activation of NK cells, and presentation of tumor-associated antigen (TAA) to tumor-specific CD8+ T cells. This was shown to be linked to CD8 T and NK cell homing to lesions (151). DCs' ability to activate NK cells can also be boosted by infecting the latter cells directly with particular OVs. Recent research has shown that the newly developed OV Maraba MG1 may directly infect and activate NK cells as well as mature DC cells. Both of these cell types are necessary for the MG1-induced reduction in postoperatively metastatic illness (152). Another research explained the oncolytic poxvirus Parapoxvirus ovis [Orf virus (OrfV)] as viral immunotherapy for ovarian cancer. The outcomes demonstrated the efficacy of OrfV as a monotherapy in a mouse model of metastatic epithelial ovarian cancer. OrfV intervention depended on NK cells, and antitumor CD8 + T-cell responses were lost when these cells were lacking. DCs are necessary for OrfV therapy, as demonstrated in studies using BATF3 knockout mice, which lack mature DCs.

Additionally, after OrfV, DCs controlled antitumor NK and T-cell responses to mediate antitumor effectiveness. OrfV was successfully paired with primary tumor excision, a frequent therapeutic option in human patients, for the best therapeutic result. Human RNA sequencing datasets analysis demonstrated that intratumoral NK cells are positively associated with survival and that DCs and NK cells are correlated in human ovarian cancer (153). For recombinant Sendai virus (rSeV), DCs and NK cells worked together in a similar way. DCs triggered by direct rSeV infection substantially reduced lung metastasis in a mouse model. While both NK and CD4+ cells are required for this preservation, NK cell activation is not. This issue shows that NK cells may be required to act as regulators instead of effectors in some DC-dependent immune responses (154). In **Table 1**, the most recent designated recombinant oncolytic viruses are listed. Some studies also considered the combined effect of recombinant OV therapy and other therapeutic approaches.

**Table 1:** Most recent recombinant OV used in the treatment of several cancer types.

OV type	Chemical carried	Model	Results	Combination	Refs
**Adenovirus**	IL-2, TNF-α	bilateral murine melanoma	TNF-α and IL-2 coding adenovirus local therapy improved the systemic response to aPD-1 therapy.	Anti-PD-1	(155)
**Vaccinia virus**	TIGIT	Mouse model	When combined with PD-1 or LAG-3 blockage, VV-scFv-TIGIT was able to completely eradicate tumors that had not responded to VV or immune checkpoint blockade monotherapy.	PD-1 and LAG-3 blockade	(156)
**Herpes Simplex virus 1**	Anti-PD-1, IL-12	murine CT26 colon adenocarcinoma	A vaccine-like reaction, the activation of antigen-specific T cell responses, the inhibition of primary tumor growth, and the prevention of growth of the contralateral untreated tumor were all effects of viral therapy that increased the overall survival rate of mice.	–	(157)
**Vaccinia virus**	manganese superoxide dismutase	C57BL/6 mice	When combined with anti-PD-L1, the administration of OVV-MnSOD further enhanced the results of anticancer therapy in mice where these monotherapy strategies failed to show any benefit.	Anti-PD-1	(158)
**Herpes Simplex virus 1**	IL-12	Pet dogs with glioma	M032 therapy had no adverse effects, and the combination of surgery and oncolytic virus therapy may have helped companion dogs with spontaneous gliomas live longer.	–	(159)
**Adenovirus**	CCL19	Gastric cancer mice model	Virotherapy has the potential to significantly boost the infiltration of CD4+ and CD8+ T lymphocytes as well as the release of IFN-γ and TNF-α in tumor tissues.	–	(160)
**Herpes Simplex virus 1**	IL-12, GM-CSF	B16-F10 murine melanoma	An engineered oncolytic HSV’s GM-CSF and IL-12 work in concert to enhance the immune response and their anticancer impacts.	–	(148)
**Adenovirus**	IL-2, TNF-α	Human tumor histocultures from urological tumor	The viruses made it possible for an anti-PD-L1 (a checkpoint inhibitor) to give full responses in all of the treated animals *in vivo* (hazard ratios against anti-PD-L1 alone 0.057 or virotherapy alone 0.067).	Anti-PD-1	(161)
**Vaccinia virus**	Anti-PD-1, GM-CSF	293T cells, HUTK-143B, CV1, Py230, B16-F10, EL4, MC38	For people living with cancer, especially those resistant to PD-1/PD-L1 blocking therapy, this modified oncolytic virus offers an effective, personalized tumor-specific oncolytic immunotherapy.	–	(162)
**Herpes Simplex virus 1**	PTEN	DB7, Met-1, MVT-1, MDA-MB-231, SK-BR-3, MCF-7, MDA-MB-468	In mice with intracranial tumors, a single treatment of HSV-P10 produced long-term survivors and primed anticancer T-cell immunity, leading to tumor refusal.	–	(163)
**Adenovirus**	bispecific T-cell engager	DLD, SKOV3, A549, HEK293A, NHDF, CHO, NHBE	Direct virolysis and endogenous T-cell activation are combined to assault stromal fibroblasts by an engineered oncolytic adenovirus that encodes a bispecific antibody, offering a multimodal therapeutic approach in a single therapeutic agent.	–	(164)
**Vaccinia virus**	ING4	Acute myeloid leukemia model	The effectiveness of the combination of oVV-ING4 and cytarabine was examined both *in vitro* and *in vivo*; it dramatically reduced leukemia cell survival *in vitro* and decreased the growth of a xenografted KG-1 AML tumor *in vivo*.	Cytarabine	(165)
**Vaccinia virus**	Smac	Pancreatic cancer	High amounts of Smac were attained together with increased cytotoxicity and potentiated apoptosis using oVVSmac.	Gemcitabine	(166)
**Vesicular stomatitis virus**	TNF-α	EMT6, CT-26, 786-0, GM38, H460, H661, Vero, SNB75	The treatments showed a new method by which cytokine-armed VSV-51 paired with LCL161 can kill tumor cells. This produced vascular collapse in solid tumors, which in turn generated a contemporaneous spike in the death rate of tumor cells.	Smac mimetic compounds	(167)

## Prospects and conclusion

So far, studies show that having a functioning anti-tumor T cell response is linked to better cancer outcomes (168). Cancer immunotherapies, which are designed to improve the establishment of anti-tumor immunity, have inspired a great deal of interest in the treatment of cancers of all different kinds. Surprisingly, the presentation of tumor antigens by DCs to tumor-specific T cells is necessary for the formation of several types of anti-tumor immune responses as well as their regulation (169). Because of this, the number of anti-cancer medications that are available in the therapeutic arsenal has significantly expanded as a direct result of immunotherapies. This article highlights the significance of NK cells and DCs as virotherapy mediators and blockers. OVs, similar to other types of immunotherapies such as immune checkpoint inhibitors and CAR, function by harnessing and activating the immune system in order to combat cancer cells. Despite the fact that immunotherapies have demonstrated promising results, there is still a lot more to understand about the intricate immune response that is involved in the process of how they work. After the success of OVs loaded with cytokines in mouse models, researchers have turned their attention to various immuno-stimulatory payloads that may be delivered to the TME. Antibodies that block checkpoint receptors are included in these payloads (170). Currently, trastuzumab, a monoclonal antibody produced by an adenovirus, is used to treat breast cancer as an example (171). Due to the fact that trastuzumab was provided in the TME at extraordinarily high concentrations as a result of OV-delivery, the NK-mediated ADCC of breast cancer cells was significantly enhanced. This article demonstrates that utilizing NK-engagers to direct NK cells toward tumors can be an efficient and successful treatment strategy when a target that is unique to the tumor is readily available (172), it is more effective than monoclonal antibodies to target NK cells, might be encoded in OVs to give novel therapeutic options in this setting.

Using NK cells and DCs as a delivery system to improve virotherapy’s efficacy NK cell-mediated Ad delivery system (Ad@NK) for combinational immunotherapy and virotherapy of cancer was developed in a recently published study by Ding et al. In this biohybrid system, tumor-homing NK cells function as bioreactors and shelters for the loading, defense, and release of Ads, resulting in a highly effective systemic tumor-targeted delivery. Ad infection provides NK cells with feedback by triggering type I interferon signaling in a STAT4granzyme B-dependent way, which enhances NK cell antitumor immunity. Additionally, it has been discovered that by encouraging dendritic cell maturation and the polarization of macrophages to the M1 phenotype, the Ad@NK system can alleviate immunosuppression in the TME. Both in vitro and in vivo investigations have shown that Ad@NKs have exceptional anticancer and antimetastatic qualities. These features are evidenced by the killing of tumor cells, the activation of immunogenic cell death, and the immunomodulation of the TME (173). The DC is also capable of delivering oncolytic reoviruses (174) and measles virus (175). In these studies, DCs internalized the virus, which protected it from being destroyed by neutralizing antibodies. Many different types of immune cells analyze this problem. However, when DCs were loaded with reovirus, strong anti-tumor as well as anti-viral immune responses were observed. This resulted in an increased survival rate of mice who had melanoma. It has been proven that therapeutic infusions of reovirus, in particular, are ineffective in previously reovirus-exposed hosts (176). Hence, using immune cells like DCs as cell carriers offers a way to improve the systemic dispersion of OVs to target primary and metastatic tumors. This is particularly useful for OVs against which it is anticipated that the host already possesses anti-viral immunity as a result of prior exposure. (177).

In order to better understand the dual properties of NK cells and DCs during OV therapy and their interaction in terms of improving cancer immunotherapy, future studies should use appropriate preclinical models to explain better the various effects of NK cells and DCs for OV treatment of tumors. In addition, clinical trials looking at OVs should look into the role of these two critical immune cells in influencing therapeutic results. Upcoming OV-based treatments, if manipulated to express transgenes or in combination with other drugs/immunotherapies, will benefit from taking into account the role of NK cells and DCs in facilitating the therapeutic response.

## Author contributions

Conceptualization, MG, NNGF and LA. Methodology, NG and NNGF. Validation, LA. Data curation, PK. Writing-original draft preparation, LG, MG, NNGF and NG. Writing-review and editing, all. All authors have read and agreed to the published version of the manuscript.
